# Use of Radiomics in Characterizing Tumor Hypoxia

**DOI:** 10.3390/ijms26146679

**Published:** 2025-07-11

**Authors:** Mohan Huang, Helen K. W. Law, Shing Yau Tam

**Affiliations:** 1 School of Medical and Health Sciences, Tung Wah College, Hong Kong SAR, China; mhhuang@twc.edu.hk; 2 Department of Health Technology and Informatics, The Hong Kong Polytechnic University, Hong Kong SAR, China; helen.law@polyu.edu.hk

**Keywords:** deep learning, machine learning, medical imaging, non-invasive assessment, tumor hypoxia, radiomics

## Abstract

Tumor hypoxia involves limited oxygen supply within the tumor microenvironment and is closely associated with aggressiveness, metastasis, and resistance to common cancer treatment modalities such as chemotherapy and radiotherapy. Traditional methodologies for hypoxia assessment, such as the use of invasive probes and clinical biomarkers, are generally not very suitable for routine clinical applications. Radiomics provides a non-invasive approach to hypoxia assessment by extracting quantitative features from medical images. Thus, radiomics is important in diagnosis and the formulation of a treatment strategy for tumor hypoxia. This article discusses the various imaging techniques used for the assessment of tumor hypoxia including magnetic resonance imaging (MRI), positron emission tomography (PET), and computed tomography (CT). It introduces the use of radiomics with machine learning and deep learning for extracting quantitative features, along with its possible clinical use in hypoxic tumors. This article further summarizes the key challenges hindering the clinical translation of radiomics, including the lack of imaging standardization and the limited availability of hypoxia-labeled datasets. It also highlights the potential of integrating radiomics with multi-omics to enhance hypoxia visualization and guide personalized cancer treatment.

## 1. Introduction

Tumor hypoxia refers to a lack of oxygen in tumor cells due to an imbalance between the demand and supply of oxygen [1]. Such a phenomenon is common in various cancers, including hepatocellular carcinoma (HCC), glioblastoma multiforme (GBM), lung cancer, and colorectal cancer. Hypoxia in HCC leads to tumor advancement and therapy resistance, while in GBM, hypoxic zones in the tumor microenvironment (TME) are associated with highly aggressive behavior as well as therapy resistance [2,3]. Lung cancer is accompanied by hypoxic areas, which are associated with poor prognosis and suboptimal responsiveness to treatments [4]. Colorectal cancer also displays hypoxic areas, contributing to enhanced metastatic potential and treatment resistance [5]. Hypoxia is a key factor in these cancers, which shows that it plays a very important role in tumor biology and provides a rationale for developing an effective therapeutic strategy.

Hypoxia influences many cancer treatment modalities. In the case of radiotherapy, the presence of adequate levels of oxygen in tissue is vital for the generation of reactive oxygen species, responsible for damaging cancer cell DNA [6]. Hypoxic conditions lessen this effect, thus leading to radioresistance [7]. Likewise, certain drugs act in an oxygen-dependent manner to induce cytotoxicity. Hypoxia alters drug metabolism and decreases drug delivery by virtue of aberrant tumor vasculature [8]. Targeted therapies, which are aimed at specific molecular pathways, may also be less effective in hypoxic settings, wherein the activation of alternative survival pathways is expected [9,10]. The challenges posed by tumor hypoxia emphasize the significance of properly understanding and addressing tumor hypoxia to maximize treatment success.

Radiomics is an emerging field that extracts a large number of quantitative features from medical images in order to unearth patterns that are often not observable by the naked eye [11]. Radiomics begins with image acquisition, followed by the segmentation of the region of interest, feature extraction, and analysis. These features can relate to aspects such as shape, texture, and intensity and provide a comprehensive characterization of tumor phenotypes [12,13]. Potential applications of radiomics in cancer research include diagnosis, prognosis, and the prediction of treatment response. Converting images into high-dimensional data allows for a non-invasive approach to assessing tumor characteristics and monitoring disease evolution [14].

The incorporation of machine learning and deep learning techniques has further broadened the horizons of radiomics in oncology. Machine learning algorithms can handle large datasets and identify complex patterns, thus increasing the accuracy of the predictions provided regarding disease outcomes [15]. Deep learning, a sub-area of machine learning, applies neural networks to model complex relationships within data, which enables the intricacies of imaging features linked to the endpoints of interest to be identified [16]. Advancements of this type have increased diagnostic accuracy and prognostic assessments, thus facilitating personalized treatment strategies. For example, deep neural networks have been studied in predicting relapses in mantle cell lymphoma using computed tomography (CT) baseline images [17,18,19].

This review summarizes recent developments in radiomics in terms of outlining tumor hypoxia and therapeutic aspects. Various imaging modalities are discussed: CT, magnetic resonance imaging (MRI), and positron emission tomography (PET) in detecting hypoxic regions inside tumors. This study also addresses the merits of coupling radiomic data with various molecular and biological means to augment targeted therapies against hypoxia. This review discusses the role of radiomics in meeting the challenges posed by tumor hypoxia while pointing out directions for inquiry in the future.

## 2. Review Methodology

This article presents a narrative review aimed at synthesizing current knowledge on the use of radiomics in characterizing tumor hypoxia. A narrative review was chosen to allow for the comprehensive coverage of both foundational concepts and recent interdisciplinary advances across imaging modalities, radiogenomics, and artificial intelligence applications. We searched PubMed, Web of Science, and Google Scholar using keywords including “tumor hypoxia”, “radiomics”, “PET”, “MRI”, “deep learning”, and “radiogenomics”. Priority was given to peer-reviewed articles published between 2014 and 2025, with an emphasis on recent high-impact studies, systematic reviews, and clinically relevant research. Although this review is not PRISMA-compliant, it follows best practices for narrative reviews as per recent reporting guidelines to ensure clarity and scientific rigor.

**Figure 1:**
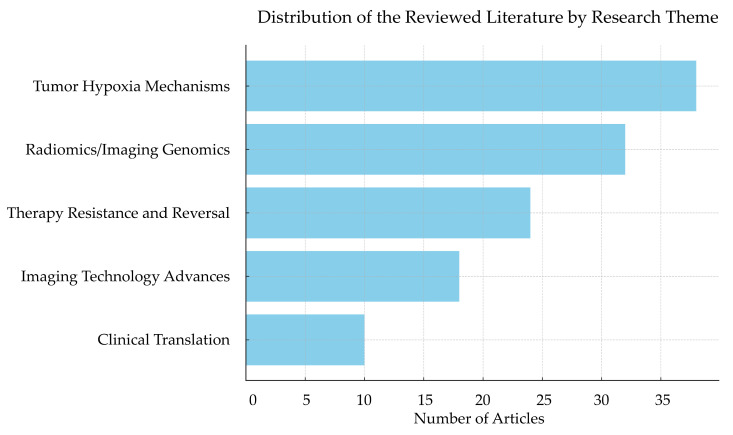
The distribution of the reviewed literature by research theme. This bar chart summarizes the distribution of reviewed articles according to five major research themes: tumor hypoxia mechanisms (38 studies, 31.1%), radiomics and imaging genomics (32 studies, 26.2%), therapy resistance and reversal strategies (24 studies, 19.7%), imaging technology advances (18 studies, 14.8%), and clinical translation/trials (10 studies, 8.2%).

In total, 122 peer-reviewed articles were included in this narrative review, categorized into five main research themes shown in Figure 1: tumor hypoxia mechanisms, radiomics and imaging genomics, therapy resistance and reversal strategies, imaging technology advances, and clinical translation/trials. Thematic analysis revealed increasing interdisciplinary attention to the integration of imaging biomarkers with biological mechanisms. Notably, this study identified two cross-disciplinary research hotspots: one is hypoxia-focused radiomics (16 studies), which predicts hypoxia-driven gene expression and metabolic phenotypes through imaging features; the other is the integrated research of hypoxia and immunotherapy (12 studies), which explores the enhancement in immune responses through microenvironmental reprogramming under hypoxic conditions. These studies indicate that radiomics is gradually emerging as a bridge connecting microenvironmental status and therapeutic strategies.

## 3. Tumor Hypoxia: Mechanisms and Clinical Implications

### 3.1. Definition and Pathophysiology of Tumor Hypoxia

Tumor hypoxia refers to a condition where the partial pressure of oxygen (pO_2_) is below 2%, impacting multiple processes in the TME [10]. This low-oxygen state occurs when the existing blood vessels cannot meet oxygen demand, which is due to the rapid proliferation and growth of cancer cells. Many solid tumors present evidence of hypoxia. In the clinical setting, hypoxia has been generally associated with a more aggressive biological behavior and resistance to therapy [20].

At the molecular level, hypoxia-inducible factors (HIFs), especially HIF-1α and HIF-2α, play pivotal roles in the adaptation of cells to low-oxygen environments. Under normoxia, HIFs are hydroxylated and subsequently degraded. However, under hypoxic conditions, they inhibit hydroxylation, leading to HIF stabilization and accumulation [21]. Following stabilization, HIF-1α mainly regulates genes that are involved in glycolysis, inducing a shift from oxidative phosphorylation to glycolysis for efficient energy generation under hypoxic conditions. In contrast, by regulating erythropoiesis and angiogenesis-associated genes, HIF-2α facilitates the formation of new blood vessels to improve oxygen supply. These differences demonstrate the complexity of HIF regulation in tumor biology [22]. This mechanistic complexity has been thoroughly reviewed in a recent work by Liao et al. [23], which provides an integrative overview of hypoxia-driven biological reprogramming, including glycolytic shift, angiogenesis, and epigenetic modulation, alongside a detailed discussion of therapeutic vulnerabilities associated with these pathways. Their synthesis highlights the multifaceted role of hypoxia in promoting tumor heterogeneity and therapy resistance, offering valuable insights for translational research. Recent insights also highlight the temporal dynamics of hypoxia response, where shifts in oxygen-sensing enzyme activity orchestrate adaptive transcriptional and epigenetic changes that contribute to tumor aggressiveness and immune evasion [24].

Hypoxia plays a critical role in tumor metabolism, the immune microenvironment, and therapeutic resistance. Metabolically, hypoxic conditions induce a shift toward anaerobic glycolysis, known as the Warburg effect [25]. This leads to an increase in lactate production and the acidification of the tumor microenvironment. The reduced pH destabilizes the extracellular matrix and turns on proteolytic enzymes, like matrix metalloproteinases, thus enhancing tumor invasion and metastasis [26]. Hypoxia has thus also been shown to upregulate immune checkpoint molecules such as programmed death ligand 1 (PD-L1) and cytotoxic T-lymphocyte-associated protein 4 (CTLA-4) under immune microenvironments. These receptors interact with their corresponding ligands expressed on T cells, inhibiting their activation and proliferation, promoting immune suppression, and eventually leading to tumor immune evasion [27]. Moreover, hypoxia also promotes genomic instability by downregulating DNA repair pathways and thus increasing mutation rates. Such genetic variability by itself allows for the selection of more aggressive and therapy-insensitive sub-populations of tumor cells. These cells may evade therapy through altered drug transporter expression or the activation of anti-apoptotic pathways, complicating treatment responses [28,29]. The recently developed open-source platform THER (Tumor Hypoxia Exploration and Research) stores extensive transcriptomic datasets. It explores the association between hypoxia-related transcriptomic signatures and the mechanisms of tumor initiation and progression, providing a scientific basis for identifying valuable biomarkers [30].

### 3.2. Methods for Assessing Tumor Hypoxia

Molecular endogenous markers such as HIF-1α, carbonic anhydrase IX (CAIX), glucose transporters 1/3 (GLUT-1/3), vascular endothelial growth factor (VEGF), and monocarboxylate transporter 4 (MCT-1) also play a role in the assessment of tumor hypoxia. These proteins are significantly upregulated under low-oxygen conditions and are typically detected using immunohistochemistry. The expression levels of these markers not only act as indirect measures of tumor hypoxia but also correlate with tumor aggressiveness and poor prognosis [31]. An elevated HIF-1α level promotes various biological processes like angiogenesis and metastasis in tumor cells. CAIX is correlated with TME acidification. It supports tumor cells to adapt to acidic conditions and increase migration and invasiveness [32]. VEGF serves its role by enabling neovascularization that supplies nutrients and oxygen to the tumor, facilitating its growth and spread. At the molecular level, VEGF functions by binding specifically to corresponding receptors to regulate endothelial cell proliferation and migration [33]. In addition to endogenous markers, exogenous markers such as pimonidazole, EF-5, and Hydroxyprobe™ are frequently used to detect tumor hypoxia through immunofluorescence or flow cytometry [34,35,36]. ELK3-51, a novel exogenous agent, has been developed to bind thiol groups under hypoxic conditions, allowing for the precise visualization and quantification of oxygen-deficient areas in tissues [36].

The assessment of tumor hypoxia is essential to understand tumor progression and to develop targeted therapies. Traditionally, polarographic oxygen electrodes were the tools of choice for measuring oxygenation in tumor tissues. This method involves inserting a needle-like electrode directly into tumor tissue to obtain localized oxygen pressure readings [37,38]. They are traditionally considered the gold standard but are also very invasive and present concerns regarding their reading accuracy. First, the method used for measuring oxygen concentration leads to some mechanical damage of the tumor tissue and many complications. Single-point measurement does not directly represent heterogeneous oxygenation across the entire tumor, thus hindering the search for patterns in regions. Fluorescence quenching is the next assessment option. It is non-invasive and employs oxygen-sensitive fluorescent probes that emit specific wavelengths of light under hypoxic conditions. An increase in oxygen concentration causes the loss of fluorescence intensity (quenching). The degree of fluorescence quenching helps in determining the exact tissue oxygen levels in addition to the localization of hypoxic areas in tumors. To summarize, fluorescence quenching is less invasive than polarographic electrodes and also provides information about the distribution of oxygen in the tumor [39,40].

**Figure 2:**
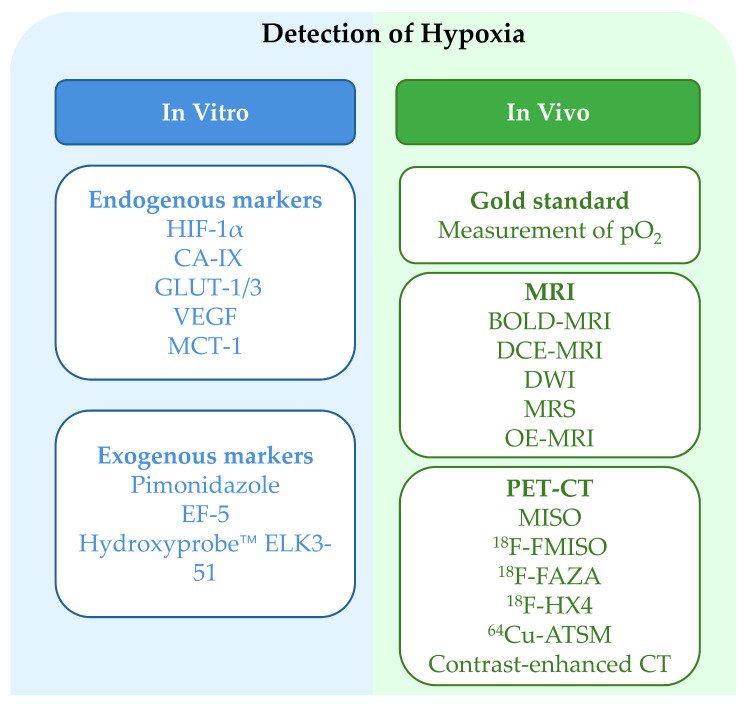
Schematic illustration of hypoxia detection approaches, encompassing in vitro and in vivo strategies. Detection methods include endogenous markers, exogenous markers, direct pO_2_ measurements, and imaging modalities such as PET and MRI [36,43,51].

Imaging modalities provide surrogate, non-invasive methods for evaluating tumor hypoxia. Techniques such as blood oxygen level-dependent (BOLD) MRI, dynamic contrast-enhanced (DCE) MRI, diffusion-weighted imaging (DWI), magnetic resonance spectroscopy (MRS), and oxygen-enhanced (OE) MRI detect the presence of poorly oxygenated tumors when assessing hemodynamic changes, blood oxygenation, and tissue perfusion [41,42,43,44,45]. Using radiotracers like misonidazole (MISO), fluorine-18 fluoromisonidazole (^18^F-FMISO), fluorine-18 fluoroazomycin arabinoside (^18^F-FAZA), ^18^F-flortanidazole (^18^F-HX4), and copper-64 diacetyl-bis (N4-methylthiosemicarbazone) (^64^Cu-ATSM), PET visualizes and quantifies intratumoral hypoxia [45,46,47]. Contrast-enhanced CT, particularly functional CT, assesses key parameters such as blood volume and blood flow for the evaluation of tumor oxygenation [48]. These imaging techniques allow for the dynamic assessment of tumor hypoxia and give critical information to physicians regarding oxygenation-correlated disease progression. In radiation treatment planning, such imaging techniques help in determining the exact irradiation fields, minimizing normal tissue damage, and optimizing dose distribution by targeting hypoxic tumor cells [49]. In targeted therapies, these imaging modalities will also allow for the selection of patients who can benefit from anti-angiogenic therapies that assist in enhancing the specificity and efficacy of cancer treatment [50]. Figure 2 summarizes the classification of tumor hypoxia detection methods, including approaches ranging from molecular marker-based methods to imaging modalities such as MRI, PET, and CT.

### 3.3. Clinical Impact of Hypoxia on Cancer Therapy

Tumor hypoxia plays an important role in clinical outcomes, especially in treatment resistance. In radiotherapy, oxygen participates in chemical reactions that ultimately produce reactive oxygen species, such as hydroxyl radicals (·OH) and superoxide anions (O_2_^−^). These reactive species are known for their relatively high oxidative reactivity and can induce DNA double-strand breakage and base damage in tumor cells [52]. However, under hypoxic conditions, the limited amount of oxygen decreases ROS generation and subsequent radiation-induced DNA damage, which contributes to significantly decreased radiosensitivity [53,54]. To achieve comparable therapeutic effects in normoxic tumors, it is necessary to escalate the dose of radiation. However, this may elevate the risk of damage to surrounding normal tissues and lead to the development of various serious complications [55].

In chemotherapy, hypoxia significantly alters tumor metabolism and contributes to drug resistance. Tumor cells under hypoxic conditions primarily depend on anaerobic glycolysis. The metabolic change alters the expression and function of transport proteins on the cell membrane, as well as physiological processes such as diminished drug uptake and the increased expression of efflux pumps. One of the involved proteins, P-glycoprotein (P-gp), belongs to the ATP-binding cassette transporter superfamily and is highly implicated in multidrug resistance [56]. Hypoxia activates several signaling pathways, including the HIF-1α pathway. Activated HIF-1α can modulate the expression, either directly or indirectly, of genes coding for P-gp and thereupon significantly upregulates P-gp at the cell membrane. P-gp utilizes the energy obtained from ATP hydrolysis to cotransport chemotherapy drugs outside of the cell against a concentration gradient, which leads to a decrease in intracellular drug concentration and consequently reduces chemotherapy’s effects [57]. Additionally, hypoxia promotes DNA damage repair mechanisms and further facilitates tumor cell resistance to chemotherapy. Key kinases such as ATM become activated under hypoxic conditions. ATM kinase initiates complex phosphorylation cascades via autophosphorylation and the phosphorylation of downstream substrates. DNA repair proteins like BRCA1 are then rapidly activated for DNA repair. The stimulated repair mechanisms enable tumor cells to repair chemotherapy-induced DNA damage and evade apoptosis, thereby supporting survival and re-population [58,59].

Hypoxia significantly contributes to tumor immune evasion by promoting the accumulation of immunosuppressive regulatory T cells (Tregs) and myeloid-derived suppressor cells (MDSCs). Tregs secrete immunosuppressive cytokines such as interleukin-10 (IL-10) and transforming growth factor beta (TGF-β), which inhibit the tumoricidal functions of cytotoxic T lymphocytes and natural killer (NK) cells [60]. MDSCs employ a variety of mechanisms to suppress immune responses. For example, they express increased levels of arginase 1 (ARG1) that consumes arginine within TME. The depletion of arginine impairs T-cell metabolism and functionality [61,62]. Hypoxia also downregulates MHC-I expression via HIF-mediated transcriptional repression, impairing tumor recognition by cytotoxic T cells and facilitating immune escape [63].

Hypoxia in the TME greatly alters the activity of M2-like tumor-associated macrophages (TAMs). M2-like TAMs predominantly express protumoral factors and behave in what can be described as a new manner; the usual M2 functions include those involved in the role of angiogenic factors like VEGF, which promotes angiogenesis, leading to more nutrients and oxygen in the tumor. MMP-2 and MMP-9 degrade extracellular matrix components, which are used for tumor invasion and metastasis promotion. They secrete immunosuppressive cytokines such as IL-10, which reduce the efficacy of immune effector cells (T cells and NK cells). Hence, the immune system is disadvantaged in recognizing and eliminating tumor cells [64].

## 4. Radiomics in Tumor Hypoxia Characterization

### 4.1. Extraction of Radiomics-Based Hypoxia Imaging Features

Radiomics is a field that entails the extraction of high-dimensional quantitative features from medical images to find patterns that correlate with tumor characteristics, including hypoxia. These features include the texture, shape, and pixel intensity distribution aspects of the tumor. Texture features quantify the spatial arrangement of the pixel intensities as an indicator of heterogeneity in the tumor. This heterogeneity represents variations at the cellular level, such as in cell composition, cellular metabolic activity, and gene expression. These variations are largely driven by changes in the TME, including irregular vascularization, poor perfusion, and hypoxic stress, which lead to spatially heterogeneous oxygen and nutrient distribution. Such microenvironmental conditions directly influence radiomic features by altering tissue density, cellular morphology, and metabolic signatures that are captured in medical imaging. Therefore, radiomic features can serve as indirect biomarkers reflecting the state of the TME, especially the presence and extent of hypoxia [65,66,67,68]. Texture analysis is employed to describe the variations in imaging data [69]. Shape features describe the geometric properties of tumors such as volume, surface area, perimeter, sphericity, and lobulation index. These parameters give an idea of tumor size and contour, which are relevant for assessing growth patterns and invasiveness. For example, lobulated tumors tend to have a greater invasive potential [70]. Intensity features assess the distribution of value across the pixels, which corresponds to the density and composition of the tumor. Different tissue components have different gray levels depending on the imaging modality [71]. A radiomic analysis of these features could identify the image correlation of hypoxic regions within tumors accurately.

**Figure 3:**
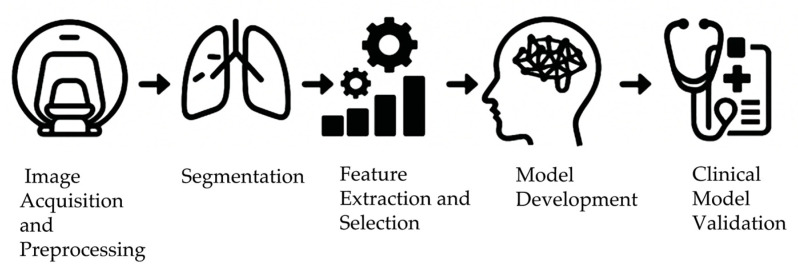
The workflow of radiomics-based tumor hypoxia characterization. The process includes image acquisition (CT, MRI, PET), preprocessing (resampling, normalization, denoising), segmentation (manual, semi-automatic, or automatic), feature extraction (e.g., texture, shape, intensity), feature selection (e.g., LASSO, mRMR), model development (e.g., machine learning, deep learning), and clinical model validation.

In radiomic research, establishing standardized workflows (Figure 3) and relevant feature selection is crucial for model robustness and reproducibility [72]. The workflow starts with image acquisition, where technical differences and scanning parameter adjustments can lead to significant variations in images from different sources. Therefore, preprocessing steps (such as resampling, normalization, and denoising) mitigate such discrepancies and enhance the comparability of subsequent analyses. For example, resampling standardizes scanning parameters like slice thickness and resolution by unifying the voxel dimensions of original medical images, ensuring each image maintains a common spatial reference to avoid minor detail loss and information bias in analysis. Normalization establishes a common range for gray values across different devices, eliminating signal differences caused by inter-device variations to build a stable and homogeneous database for subsequent analysis. Denoising removes the noise introduced during acquisition, making standardized images more suitable for feature extraction [73]. Currently, publicly available databases such as The Cancer Imaging Archive (TCIA) provide standardized imaging data linked with clinical and genomic information, which are widely used in radiomic studies to investigate tumor characteristics, including hypoxia [74,75].

Segmentation then delineates regions of interest (ROIs) within tumors, either manually or through (semi-)automated techniques. Manual segmentation remains the gold standard in terms of anatomical accuracy but is time-consuming and subject to inter-observer variability [76]. To address these limitations, a variety of semi-automated and fully automated segmentation tools have been developed. Widely used software programs such as 3D Slicer and ITK-SNAP offer interactive semi-automatic segmentation based on thresholding, region-growing, or active contour models [77,78]. For fully automated approaches, deep learning-based models, especially U-Net architectures, have demonstrated superior performance [79].

This is followed by feature extraction, for which a variety of computational tools have been developed. One of the most widely used open-source platforms is PyRadiomics, a Python-based toolkit that allows for the high-throughput extraction of quantitative imaging features from medical images [80]. It supports feature extraction from 2D/3D medical images, including first-order statistics, shape, and texture features derived from matrices such as the gray-level co-occurrence matrix (GLCM), gray-level run length matrix (GLRLM), and gray-level size zone matrix (GLSZM). It also can provide reproducible standardized preprocessing workflows. PyRadiomics complies with the Imaging Biomarker Standardization Initiative (IBSI), promoting consistency across studies. Its extensibility and integration with preprocessing workflows have made it a standard tool in radiomic studies. In addition to PyRadiomics, several open-source extensions and platforms have been developed to enhance radiomic workflows. For example, Py-rex (https://github.com/zhenweishi/Py-rex) (accessed on 8 July 2025) extends PyRadiomics’ functionalities to support the direct input of DICOM and RTSTRUCT files [81]. PyRadiomics-based glioma grading provides a complete pipeline for extracting radiomic features and building machine learning models to classify glioma grades [82]. A wide range of open-source tools specifically dedicated to tumor hypoxia assessment can be found at https://github.com/search?q=tumor%20hypoxia&type=repositories (accessed on 8 July 2025). These resources greatly enhance reproducibility, cross-study comparison, and translational value in tumor hypoxia research. A summarized list of relevant repositories is provided in Table 1.

Feature selection has its own importance in the framework of radiomic analysis. Minimum Redundancy Maximum Relevance (mRMR) and Least Absolute Shrinkage and Selection Operator (LASSO) are the most widely used techniques. mRMR identifies features highly correlated with hypoxia status while minimizing redundancy. This ensures that selected features are both informative and non-overlapping, improving model clarity and performance [83,84]. LASSO, on the other hand, constructs a linear model with an L1 regularization term to compress and select feature coefficients. In this way, features less associated with hypoxia are assigned coefficients closer to zero and eventually eliminated. This decreases the dimensionality of the data while retaining the most relevant features pertaining to tumor hypoxia status [85,86]. These feature selection methods will help improve analytical efficiency and accuracy for the construction of imaging-based predictive models of tumor hypoxia.

Finally, selected features are used to build predictive models through machine learning (ML) or deep learning (DL) algorithms. Machine learning (ML) techniques are pivotal in the radiomics-based prediction of tumor hypoxia. Supervised learning methods, such as support vector machines (SVMs), random forests, and extreme gradient boosting (XGBoost), are frequently employed to develop predictive models. SVMs identify optimal hyperplanes to effectively distinguish between different hypoxic states in tumor data. Random forests, as ensemble learning algorithms, construct multiple decision trees to enhance model stability and accuracy [87,88]. XGBoost utilizes efficient gradient boosting to process large-scale data and capture complex nonlinear relationships [89]. These models iteratively learn from labeled tumor images and corresponding hypoxia annotations, enabling accurate oxygenation status classification.

Deep learning (DL) techniques, particularly convolutional neural networks (CNNs), have also found their place within radiomics. CNNs automatically extract hierarchical feature representations from imaging data, varying from crude features, such as edges and textures, to features complex in terms of their implications, which is relevant to the state of tumor hypoxia [73,90]. This end-to-end learning framework reduces reliance on manual feature engineering and captures intricate patterns often missed by traditional methods, facilitating a deeper understanding of hypoxia expression in medical imaging.

Several ML and DL models are using tumor hypoxia to predict patient outcomes. For instance, there is a DL-based radiomic approach to predicting early tumor regression in head and neck cancers. Pre-treatment imaging data have been used to foresee responses to radiotherapy [91]. In breast cancer research, DL models have been employed to identify morphological features associated with hypoxia in histopathological images, investigating traits such as cell density, nuclear morphology, and vascular distribution for precise hypoxic delineation in tumor tissue [92]. Through a deep analysis of large medical image datasets, such models provide greater efficiency and credibility in tumor hypoxia prediction.

Despite these advances, clinical model validation is still required to demonstrate applicability for tumor hypoxia detection. The foremost challenge is the large number of required annotated datasets to train strong models. A small sample size for training models leads to overfitting and the non-generalizability of models. In addition, variability in imaging protocols across institutions introduces inconsistencies and decreases reproducibility in the results [93]. To address this, researchers are focused on validating imaging procedures to reduce variability across datasets and improve applicability to models. Additionally, the development of explainable artificial intelligence (XAI) is ongoing, aiming to improve model transparency, build clinician trust, and facilitate clinical integration [94].

### 4.2. Application of Radiomics in Tumor Hypoxia Characterization

Radiomics is a widely utilized field for assessing tumor hypoxia across diverse imaging modalities. Within the sphere of MRI-based radiomics, techniques such as dynamic contrast-enhanced MRI (DCE-MRI) and blood oxygen level-dependent MRI (BOLD-MRI) have been employed to extract features relating to tumor perfusion and oxygenation. DCE-MRI assesses contrast agent kinetics and, in turn, tumor vasculature. After intravenous (IV) injection, the contrast agent enters cancerous tissues via blood vessels, and DCE-MRI successfully captures the rates of influx, distribution, and efflux. These measurements give vital insights into angiogenesis, vascular density, and permeability, all important indicators of tumor aggressiveness and growth potential [95]. On the other hand, BOLD-MRI gives some indication of tissue oxygenation by monitoring the deoxyhemoglobin level in the given tissue. The level of deoxyhemoglobin increases when the concentration of oxygen decreases. This change alters magnetic susceptibility within these tissues, and BOLD-MRI can sense such changes and map hypoxic tumor regions. Imaging offers crucial evidence for tumor hypoxia assessment [96,97]. Advanced MRI sequences, including T2-weighted imaging (T2WI) and diffusion-weighted imaging (DWI), have shown great potential for the non-invasive mapping of hypoxia. A previous study incorporated T2WI texture features and apparent diffusion coefficient (ADC) maps generated via DWI to build a hypoxia prediction model for glioblastoma. This model was strongly correlated with invasive HIF-1α immunohistochemistry results (AUC = 0.89). This method enables the spatial representation of the intratumoral hypoxic subregions in a treatment plan, increasing the capability for judging the distribution and extent of hypoxia [98]. Most importantly, it paves the way for individualized radiotherapy dose escalation strategies. By accurately aiming at areas which are hypoxic, the effectiveness of radiotherapy can be greatly enhanced to benefit treatment outcomes. Multiparametric MRI has also been evaluated for its utility in predicting the characteristics of brain tumor hypoxia. By integrating these multiple MRI parameters, this facilitates an understanding of different and sometimes complementary relationships between different modalities, contributing to model construction and resulting in a more accurate prediction of hypoxic tumors [99]. Such models help physicians in different treatment planning procedures in determining the exact delineation of irradiation margins to allow for dose amplification before therapy. The dynamics of hypoxia when conducting such treatment can be monitored in real time, allowing for the customized modification of treatments. This provision would help optimize treatment efficacy and safety. In addition, several studies have made attempts to correlate MRI-derived radiomic features with hypoxia-associated biomarkers. The purpose of this is to establish non-invasive imaging biomarkers for determining tumor hypoxia reliably. If validated, these biomarkers could reduce the need for invasive procedures, decreasing patient discomfort and associated medical risks.

**Figure 4:**
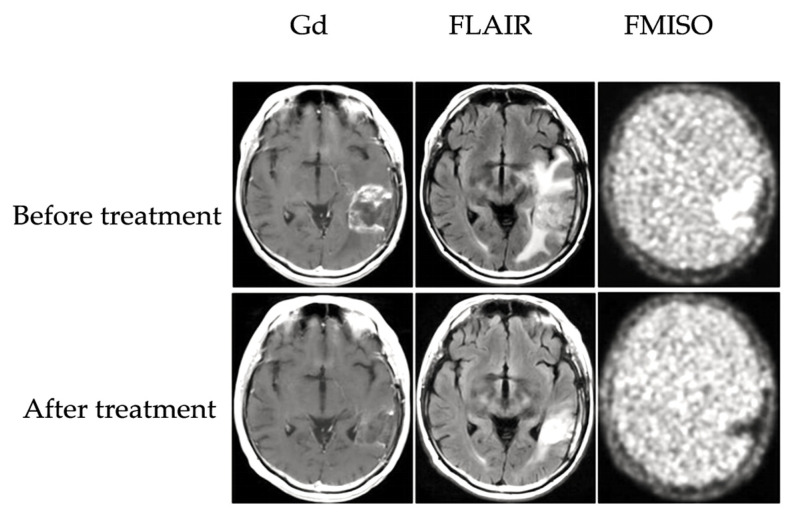
Representative MRI and FMISO-PET images of a recurrent glioma patient before and after bevacizumab treatment. Gadolinium-enhanced MRI and FLAIR MRI show morphological changes, while FMISO-PET highlights hypoxic regions with decreased tracer uptake after treatment. Adapted from Hanley R et al. [55].

Under PET-based radiomics, radiotracers such as ^18^F-FMISO and ^18^F-FAZA are employed to image tumor hypoxic regions. The distinct biochemical characteristics of these tracers lead to differential distribution and uptake in tumor tissues based on local oxygen levels. After IV injection, the tracers bind selectively to specific intracellular biomolecules and accumulate in the hypoxic TME. Signals are captured and visualized by a PET scanner. The size, extent, and exact localization of the hypoxic areas within the tumor are precisely quantifiable from the radiomic features of PET scans [100,101,102]. Novel multimodal imaging strategies that would improve the accuracy of PET radiomics in detecting tumor hypoxia have also been investigated recently. Integrating PET radiomic features with those from other imaging modalities, such as MRI and CT, has shown promise. MRI visualizes the soft tissue structure very well, while CT provides an excellent depiction of the anatomical morphology and bony structure. PET radiomics coupled with other imaging modalities allows for an integrated, multi-dimensional representation of tumors, which dramatically improves hypoxia detection. In addition, it is also demonstrated that PET-derived radiomic parameters could act as prognostic biomarkers. Certain radiomic features provide correlations between prognosis and clinical outcomes such as survival and recurrence measures. As shown in Figure 4, combining FMISO-PET and MRI can help non-invasively evaluate hypoxia dynamics and treatment response in recurrent glioma patients. This representative image illustrates pre- and post-treatment changes in gadolinium-enhanced (Gd-enhanced) MRI, Fluid-Attenuated Inversion Recovery Sequence (FLAIR), and FMISO uptake in responders to bevacizumab therapy [55]. This demonstrates their ability as accurate prognostic indicators before treatment initiation [103].

CT radiomics also has promising potential for the evaluation of tumor hypoxia. Perfusion CT imaging measures intratumoral blood flow, and a radiomic analysis of the corresponding images can yield a profile suggestive of hypoxic conditions. For instance, texture analysis can detect perfusion heterogeneity, which may correlate with hypoxic regions. Localized hypoxia alters blood perfusion, producing distinct texture patterns in the affected regions. The features of these textures are analyzed in detail by using appropriate methods like the gray-level co-occurrence matrix and run length matrix. This reveals perfusion heterogeneity for the detection of hypoxic regions in tumors. In addition, radiomic features extracted from CT images are related to tumor heterogeneity and the status of oxygenation [104]. Certain radiomic features derived from CT enhance the assessment of tumor hypoxia and enable the non-invasive evaluation of hypoxic status. Research has been performed on whether CT radiomic features can be integrated with clinical data to improve the prediction of hypoxia. These features represent tissue density and enhancement patterns for indicating the oxygenation state of the tumor and describing aspects regarding hypoxia [105]. To further improve the prediction of hypoxia-related outcomes, researchers are actively investigating a comprehensive analysis technique to integrate CT-based radiomic features with clinical data [106]. Thus, with integrated datasets and the employment of some advanced techniques such as ML and DL, the classification of patients concerning hypoxic status can be further enhanced. In summary, each modality of radiomics has unique advantages and limitations in hypoxia assessment. For a clear comparison, we summarize the imaging characteristics and radiomics-related implications of these modalities in Table 2. Finally, these approaches help in disease severity determination and prognostication over a population of patients. The hypoxia-related radiomic features employed in various studies are summarized in Table 3.

### 4.3. Application of Radiogenomics in Tumor Hypoxia

Radiogenomics, an interdisciplinary field combining radiomics with genomics and biomarker data, has opened new opportunities in understanding tumor biology, specifically tumor hypoxia [66]. It aims to discover the relationship between imaging features and molecular profiles, where the imaging biomarker may reflect underlying genetic alterations. Recent studies have shown that radiogenomics has great potential in relating specific radiomic features to the expression of hypoxia-related genes, thereby allowing one to infer tumor hypoxia levels non-invasively. This integrated approach of combining imaging along with molecular information presents a tremendous opportunity to build integrated models for the quantitative assessment of tumor hypoxia, which can dramatically turn the tide in favor of accuracy and ease of diagnosis. This enables clinicians to use the imaging and genetic information of patients to provide a true evaluation of the tumor hypoxia level and guide optimal treatment strategies [121,122]. For instance, altering doses of radiotherapy on the basis of tumor hypoxia or the correct selection of suitable targeted therapy may improve the treatment outcome and the quality of life of patients. Tumor hypoxia affects radiosensitivity, while genetic information helps to identify effective targeted agents. The suitable treatment selected will allow for the better control of tumors while sparing patients from normal organ side effects.

## 5. Clinical Applications of Radiomics in Tumor Hypoxia

### 5.1. Clinical Translation and Therapeutic Optimization

Tumor hypoxia plays a pivotal role in developing resistance to radiotherapy; therefore higher doses are needed for successful treatment. Radiomics can screen such tumors for their respective hypoxic subregions by examining various imaging characteristics like texture and intensity patterns. In radiotherapy planning, PET/MRI radiomic data can be used to delineate intratumoral hypoxic areas, enabling targeted dose escalation and an improved treatment outcome. This mode of delivering an increased dose to resistant subregions is known as “dose painting” [123]. Studies have indicated that integrating radiomics into radiotherapy planning to treat hypoxic regions can improve the chances of controlling local tumors. Preclinical studies have shown that combining DCE-MRI with ^18^F-FMISO PET can effectively assess tumor perfusion and hypoxia. In a prostate tumor model, Cho et al. demonstrated that early tracer uptake was correlated with vascular characteristics, supporting the integration of hypoxia imaging into dose painting and personalized radiotherapy planning [124]. For example, research using electron paramagnetic resonance oxygen imaging (EPROI) has successfully localized hypoxic regions for dose escalation, demonstrating significant improvements in tumor control probability [125]. Radiomics may also examine tumor hypoxia dynamics by assessing pre- and post-treatment images, thus providing insight into the changes taking place within the TME [126].

Apart from radiotherapy, radiomics assists with hypoxia-targeted therapeutics: hypoxia-activated prodrugs (HAPs) and HIF inhibitors [127]. The imaging-based identification of hypoxic areas enables the delivery of HAPs specifically to oxygen-deprived regions, thereby minimizing off-target effects in normoxic tissues. Moreover, radiomic features may correlate with HIF expression levels, supporting the personalized selection of HIF inhibitors. Targeting the HIF pathway has been associated with tumor growth inhibition and enhanced therapeutic outcomes. To further improve hypoxia-targeted drug development, radiomics can be integrated with genomic data to identify actionable targets such as HIFs and CAIX and guide rational drug design [128].

Radiomics-based hypoxia assessment is progressing toward clinical implementation, with several ongoing clinical trials exploring its utility. For example, Sanduleanu et al. developed and externally validated radiomic signatures derived from CT and FDG-PET imaging to non-invasively predict tumor hypoxia in head and neck cancer patients. Their study demonstrated strong performance and generalizability, highlighting the potential for radiomics to serve as a clinically applicable biomarker for hypoxia stratification [129]. Evofosfamide (TH-302), a hypoxia-activated prodrug, is under investigation in phase 2 trials for glioblastoma [130]. Moreover, randomized dose escalation trials using PET-guided radiomics in head and neck cancer are registered on ClinicalTrials.gov (NCT02352792) [131]. However, despite some progress shown in early-stage data, no radiomic tools for tumor hypoxia have been FDA-cleared or CE-marked yet. Regulatory challenges include the standardization of image acquisition and feature extraction, the reproducibility of models, and the lack of prospective validation. Addressing these issues through multi-center studies and harmonized pipelines is essential for future clinical integration.

### 5.2. Radiomics-Based Monitoring of Hypoxia Treatment Response

Tumor hypoxia significantly alters the TME, contributing to immunosuppression and resistance to therapies such as immunotherapy and chemotherapy. Radiomics can non-invasively assess hypoxia-induced alterations in the TME by analyzing features related to angiogenesis, perfusion heterogeneity, and tissue morphology. For example, specific radiomic signatures have been associated with immune cell infiltration, which may inform the use of immune checkpoint inhibitors, such as PD-1/PD-L1 inhibitors [132]. In addition to guiding treatment selection, radiomics also offers a powerful tool for the dynamic monitoring of treatment response. By analyzing serial images before and after therapy, radiomics can capture spatial and temporal changes in tumor hypoxia, allowing for the early detection of treatment failure or resistance. Parameters such as changes in texture, shape, and vascular features can indicate evolving hypoxic profiles and treatment-induced remodeling within the TME. To enhance monitoring precision, integrative approaches combining radiomics with liquid biopsy data, such as ctDNA and exosomal biomarkers, are being explored. While radiomics encodes spatial and structural information on hypoxia, liquid biopsies provide complementary real-time molecular-level insights. The integration of these modalities has shown promise in improving the prediction of therapeutic outcomes in hypoxia-associated cancers [133,134]. Notably, correlations between radiomic features and ctDNA levels have been reported, enabling the earlier detection of emerging therapeutic resistance and informing timely treatment adaptation [134].

### 5.3. Integrating Radiomics and Clinical Data for Personalized Treatment

Integrating clinical data with radiomic features has become pivotal in achieving personalized cancer therapy, especially in research focused on tumor hypoxia. Variables such as age, sex, and Karnofsky Performance Status (KPS) and radiomic features are commonly combined to improve prognostic models. For example, a deep learning model that predicts survival outcomes non-invasively in patients with non-small-cell lung cancer (NSCLC) by integrating electronic health record (EHR) data, radiomic data, and clinical information has been proposed. This integration facilitates a more comprehensive understanding of patient prognosis. Combined clinical and radiomic data have been shown to improve predictive accuracy. Research indicates that the fusion of imaging features with clinical and genomic data provides quantitative and objective support for cancer detection and therapeutic decision-making [135]. Multimodal approaches increase the precision of outcome predictions. They also play a pivotal role in identifying which patients are likely to benefit from hypoxia-targeted therapies, such as anti-angiogenic treatments or the use of hypoxia-activated prodrugs. Radiomics can assess tumor hypoxia through image analysis and thus facilitate patient stratification. Personalized treatment could be planned from this stratification.

Integrating radiomic data with clinical information increases the prediction of immunotherapy responses. Combining radiomics with artificial intelligence (AI) enables oncologists to forecast individual lung cancer patient responses to immunotherapy. This would help to identify which patients are likely to benefit from such treatments. As immunotherapy response is altered by heterogeneous demographic factors, combining clinical information with radiomic data could achieve more accurate assessments of treatment responses [136].

## 6. Challenges and Future Perspectives

Radiomics has become a promising tool in clinical oncology for assessing tumor hypoxia. Nevertheless, several challenges must be addressed before its routine clinical implementation.

A primary concern is the absence of standardized imaging protocols across institutions. Scanning parameters can vary, and the features extracted from images may be inconsistent due to differences in image preprocessing, acquisition, and the reconstruction of images. Studies have demonstrated that non-uniform imaging procedures compromise radiomic feature stability, which may impair model development. In addition, the radiomic workflow requires standardization. Variations in image preprocessing, feature extraction processes, and statistical methods can lead to inconsistent results. Clearly, standardized protocols are needed to ensure that radiomic features are reliable and comparable across studies [137].

The second major hurdle is the low availability of annotated hypoxia data. Many studies use a modest, single-institution dataset, constraining the robustness and applicability of radiomic models. Without the availability of large-scale and multi-center datasets that guarantee standardized imaging and detailed clinical annotations, the comprehensive validation and clinical application of radiomics remain constrained. Given that multi-center large-scale data can better encapsulate tumor hypoxia variations, they offer richer samples for training models. Currently, this sort of data insufficiency hinders models from being efficiently tested in clinical validation [137].

For the future goal of using hypoxia-targeted treatment via radiomics, the integration of multiple imaging modalities such as CT, MRI, and PET could enhance the characterization of tumor hypoxia [103,104]. Each modality presents different advantages: CT displays detailed anatomy, MRI allows for superior soft tissue contrast, and PET highlights metabolic activity. The fusion of these imaging techniques can increase accuracy in hypoxia assessment. Previous studies have demonstrated that multimodal imaging methodologies offer better predictive value for tumor hypoxia than single-modality approaches. MRI and PET data enabled accurate predictions of hypoxic areas within tumors to be made, which allows for better treatment planning. Integrating radiomics with further omics, like genomics and transcriptomics, renders a more holistic understanding of tumor biology [73]. Deep learning algorithms can handle high-dimensional data and unveil complex relationships, thus enhancing prediction abilities.

## 7. Conclusions

Radiomics has emerged as a central tool in evaluating tumor hypoxia, providing a non-invasive approach to characterizing the TME. The extraction of quantitative features from medical images leads to the identification of hypoxic regions within tumors for formulating precise treatment strategies. The heterogeneity of hypoxia across distinct tumor regions can dramatically alter treatment results. Therefore, accurate identification leads to better treatment results. Future studies should emphasize the standardization of radiomic analysis workflows across various imaging modalities and institutions to elevate the reproducibility and reliability of results. The establishment of standardized guidelines is anticipated for an easier comparison of results. Integrating radiomics with other omics approaches, like genomics and transcriptomics, may provide a more comprehensive understanding of individual tumors. This multi-omics integration could contribute greatly to advancing personalized cancer therapy.

## Figures and Tables

**Table 1:** Open-source radiomic toolkits for tumor hypoxia characterization.

Tool Name	Description	URL
pyradiomics	Feature extraction from medical images (2D/3D)	https://github.com/AIM-Harvard/pyradiomics (accessed on 8 July 2025)
Py-rex (Version 2.1)	Radiomic extension supporting DICOM/RTSTRUCT	https://github.com/zhenweishi/Py-rex (accessed on 8 July 2025)
Pyadiomics-based glioma grading	Glioma grading workflow based on PyRadiomics feature extraction	https://github.com/adhaka3/Pyadiomics-based-glioma-grading (accessed on 8 July 2025)

**Table 2:** Comparative summary of radiomics-applicable imaging modalities for tumor hypoxia assessment.

Modality	Advantages	Disadvantages	References
MRI	Functional sequences (DWI, BOLD) related to hypoxia, excellent soft tissue contrast	Susceptible to motion/artifacts, variable protocols, very long scan times	[98,107,108,109]
PET	Direct hypoxia imaging with specific tracers, limitless penetration depth	Expensive, lower spatial resolution, high ionizing radiation	[110,111,112]
CT	High spatial resolution, widely applied in clinical and preclinical settings	High ionizing radiation, suboptimal contrast between tissues, inability to provide functional data	[108,113,114,115]

**Table 3:** Summary of hypoxia feature types in radiomics.

Feature Names	Radiomic Type	References
Volume of Voxels with Hounsfield Unit (HU) > 70 within Low-Standardized Uptake Value (SUV) Subvolume, Long-Run High Gray-Level Emphasis Along Direction with Maximum Value within High-SUV Subvolume	Contrast-Enhanced CT	[116]
90th Percentile of Standardized SUV Distribution, Skewness of SUV Distribution	^18^F-FMISO PET	[116]
Gray-Level Co-Occurrence Matrix Inverse Difference (GLCM Inverse Difference)	CT	[117]
Low Gray-Level Zone Emphasis (LGZE), Classification Parameter (CP)	^18^F-FMISO PET	[118]
Tumor-to-Blood Maximum Ratio (T/Bmax), Hypoxic Volume (HV), Peak of SUV (SUVpeak)	^18^F-FMISO PET	[119]
b-value of 200 s/mm^2^ (b200), Apparent Diffusion Coefficient (ADC)	DWI MRI	[98]
Histogram-Based, Gray-Level Co-Occurrence Matrix (GLCM)	Biparametric MRI	[120]

## References

[1] Simoes-Sousa S., Littler S., Thompson S.L., Minshall P., Whalley H., Bakker B., Belkot K., Moralli D., Bronder D., Tighe A. (2018). The p38alpha Stress Kinase Suppresses Aneuploidy Tolerance by Inhibiting Hif-1alpha. Cell Rep..

[2] Feldman L. (2024). Hypoxia within the glioblastoma tumor microenvironment: A master saboteur of novel treatments. Front. Immunol..

[3] Bao M.H., Wong C.C. (2021). Hypoxia, Metabolic Reprogramming, and Drug Resistance in Liver Cancer. Cells.

[4] Guo Y., Wu M., Zhao J., Li Y. (2014). Advances in hypoxia microenvironment and chemotherapy-resistant of lung cancer. Zhongguo Fei Ai Za Zhi.

[5] Xu K., Zhan Y., Yuan Z., Qiu Y., Wang H., Fan G., Wang J., Li W., Cao Y., Shen X. (2019). Hypoxia Induces Drug Resistance in Colorectal Cancer through the HIF-1alpha/miR-338-5p/IL-6 Feedback Loop. Mol. Ther..

[6] Rockwell S., Dobrucki I.T., Kim E.Y., Marrison S.T., Vu V.T. (2009). Hypoxia and radiation therapy: Past history, ongoing research, and future promise. Curr. Mol. Med..

[7] Harrison L., Blackwell K. (2004). Hypoxia and anemia: Factors in decreased sensitivity to radiation therapy and chemotherapy?. Oncologist.

[8] Chowdhury M., Das P.K. (2024). Hypoxia: Intriguing Feature in Cancer Cell Biology. ChemMedChem.

[9] Sun X., Lv X., Yan Y., Zhao Y., Ma R., He M., Wei M. (2020). Hypoxia-mediated cancer stem cell resistance and targeted therapy. Biomed. Pharmacother..

[10] Bigos K.J., Quiles C.G., Lunj S., Smith D.J., Krause M., Troost E.G., West C.M., Hoskin P., Choudhury A. (2024). Tumour response to hypoxia: Understanding the hypoxic tumour microenvironment to improve treatment outcome in solid tumours. Front. Oncol..

[11] Rogers W., Thulasi Seetha S., Refaee T.A.G., Lieverse R.I.Y., Granzier R.W.Y., Ibrahim A., Keek S.A., Sanduleanu S., Primakov S.P., Beuque M.P.L. (2020). Radiomics: From qualitative to quantitative imaging. Br. J. Radiol..

[12] Liu Z., Wang S., Dong D., Wei J., Fang C., Zhou X., Sun K., Li L., Li B., Wang M. (2019). The Applications of Radiomics in Precision Diagnosis and Treatment of Oncology: Opportunities and Challenges. Theranostics.

[13] Li S., Zhou B. (2022). A review of radiomics and genomics applications in cancers: The way towards precision medicine. Radiat. Oncol..

[14] Zhang Y.P., Zhang X.Y., Cheng Y.T., Li B., Teng X.Z., Zhang J., Lam S., Zhou T., Ma Z.R., Sheng J.B. (2023). Artificial intelligence-driven radiomics study in cancer: The role of feature engineering and modeling. Mil. Med. Res..

[15] Avanzo M., Wei L., Stancanello J., Vallieres M., Rao A., Morin O., Mattonen S.A., El Naqa I. (2020). Machine and deep learning methods for radiomics. Med. Phys..

[16] Parekh V.S., Jacobs M.A. (2019). Deep learning and radiomics in precision medicine. Expert. Rev. Precis. Med. Drug Dev..

[17] Lisson C.S., Lisson C.G., Mezger M.F., Wolf D., Schmidt S.A., Thaiss W.M., Tausch E., Beer A.J., Stilgenbauer S., Beer M. (2022). Deep Neural Networks and Machine Learning Radiomics Modelling for Prediction of Relapse in Mantle Cell Lymphoma. Cancers.

[18] Park J.E., Kickingereder P., Kim H.S. (2020). Radiomics and Deep Learning from Research to Clinical Workflow: Neuro-Oncologic Imaging. Korean J. Radiol..

[19] Huynh B.N., Groendahl A.R., Tomic O., Liland K.H., Knudtsen I.S., Hoebers F., van Elmpt W., Malinen E., Dale E., Futsaether C.M. (2023). Head and neck cancer treatment outcome prediction: A comparison between machine learning with conventional radiomics features and deep learning radiomics. Front. Med..

[20] Muz B., de la Puente P., Azab F., Azab A.K. (2015). The role of hypoxia in cancer progression, angiogenesis, metastasis, and resistance to therapy. Hypoxia.

[21] Bae T., Hallis S.P., Kwak M.K. (2024). Hypoxia, oxidative stress, and the interplay of HIFs and NRF2 signaling in cancer. Exp. Mol. Med..

[22] Huang M., Yang L., Peng X., Wei S., Fan Q., Yang S., Li X., Li B., Jin H., Wu B. (2020). Autonomous glucose metabolic reprogramming of tumour cells under hypoxia: Opportunities for targeted therapy. J. Exp. Clin. Cancer Res..

[23] Liao C., Liu X., Zhang C., Zhang Q. (2023). Tumor hypoxia: From basic knowledge to therapeutic implications. Semin. Cancer Biol..

[24] Lee S.C.S., Pyo A.H.A., Koritzinsky M. (2023). Longitudinal dynamics of the tumor hypoxia response: From enzyme activity to biological phenotype. Sci. Adv..

[25] Burns J.S., Manda G. (2017). Metabolic Pathways of the Warburg Effect in Health and Disease: Perspectives of Choice, Chain or Chance. Int. J. Mol. Sci..

[26] Lopez-Lazaro M. (2008). The warburg effect: Why and how do cancer cells activate glycolysis in the presence of oxygen?. Anticancer. Agents Med. Chem..

[27] Ma S., Zhao Y., Lee W.C., Ong L.T., Lee P.L., Jiang Z., Oguz G., Niu Z., Liu M., Goh J.Y. (2022). Hypoxia induces HIF1alpha-dependent epigenetic vulnerability in triple negative breast cancer to confer immune effector dysfunction and resistance to anti-PD-1 immunotherapy. Nat. Commun..

[28] Kaplan A.R., Glazer P.M. (2020). Impact of hypoxia on DNA repair and genome integrity. Mutagenesis.

[29] Chen Z., Han F., Du Y., Shi H., Zhou W. (2023). Hypoxic microenvironment in cancer: Molecular mechanisms and therapeutic interventions. Signal Transduct. Target. Ther..

[30] Zhang Y., Lin A., Yang H., Liu Z., Cheng Q., Zhang J., Luo P. (2023). THER: Integrative Web Tool for Tumor Hypoxia Exploration and Research. bioRxiv.

[31] Kuijper A., van der Groep P., van der Wall E., van Diest P.J. (2005). Expression of hypoxia-inducible factor 1 alpha and its downstream targets in fibroepithelial tumors of the breast. Breast Cancer Res..

[32] Lee S.H., Griffiths J.R. (2020). How and Why Are Cancers Acidic? Carbonic Anhydrase IX and the Homeostatic Control of Tumour Extracellular pH. Cancers.

[33] Zanetti J.S., Soave D.F., Oliveira-Costa J.P., da Silveira G.G., Ramalho L.N., Garcia S.B., Zucoloto S., Ribeiro-Silva A. (2011). The role of tumor hypoxia in MUC1-positive breast carcinomas. Virchows Arch..

[34] Aguilera K.Y., Brekken R.A. (2014). Hypoxia Studies with Pimonidazole in vivo. Bio-Protocol.

[35] Lee J., Siemann D.W., Koch C.J., Lord E.M. (1996). Direct relationship between radiobiological hypoxia in tumors and monoclonal antibody detection of EF5 cellular adducts. Int. J. Cancer.

[36] Godet I., Doctorman S., Wu F., Gilkes D.M. (2022). Detection of Hypoxia in Cancer Models: Significance, Challenges, and Advances. Cells.

[37] Shaw A.D., Li Z., Thomas Z., Stevens C.W. (2002). Assessment of tissue oxygen tension: Comparison of dynamic fluorescence quenching and polarographic electrode technique. Crit. Care.

[38] Walsh J.C., Lebedev A., Aten E., Madsen K., Marciano L., Kolb H.C. (2014). The clinical importance of assessing tumor hypoxia: Relationship of tumor hypoxia to prognosis and therapeutic opportunities. Antioxid. Redox Signal.

[39] Abou Khouzam R., Janji B., Thiery J., Zaarour R.F., Chamseddine A.N., Mayr H., Savagner P., Kieda C., Gad S., Buart S. (2023). Hypoxia as a potential inducer of immune tolerance, tumor plasticity and a driver of tumor mutational burden: Impact on cancer immunotherapy. Semin. Cancer Biol..

[40] Luo W., Wang Y. (2019). Hypoxia Mediates Tumor Malignancy and Therapy Resistance. Adv. Exp. Med. Biol..

[41] Rickard A.G., Palmer G.M., Dewhirst M.W. (2019). Clinical and Pre-clinical Methods for Quantifying Tumor Hypoxia. Adv. Exp. Med. Biol..

[42] Tsien C., Cao Y., Chenevert T. (2014). Clinical applications for diffusion magnetic resonance imaging in radiotherapy. Semin. Radiat. Oncol..

[43] Glunde K., Jiang L., Moestue S.A., Gribbestad I.S. (2011). MRS and MRSI guidance in molecular medicine: Targeting and monitoring of choline and glucose metabolism in cancer. NMR Biomed..

[44] Dubec M.J., Price J., Berks M., Gaffney J., Little R.A., Porta N., Sridharan N., Datta A., McHugh D.J., Hague C.J. (2024). Oxygen-Enhanced MRI Detects Incidence, Onset, and Heterogeneity of Radiation-Induced Hypoxia Modification in HPV-Associated Oropharyngeal Cancer. Clin. Cancer Res..

[45] Fleming I.N., Manavaki R., Blower P.J., West C., Williams K.J., Harris A.L., Domarkas J., Lord S., Baldry C., Gilbert F.J. (2015). Imaging tumour hypoxia with positron emission tomography. Br. J. Cancer.

[46] Gouel P., Decazes P., Vera P., Gardin I., Thureau S., Bohn P. (2023). Advances in PET and MRI imaging of tumor hypoxia. Front. Med..

[47] Chapman J.D. (1979). Hypoxic sensitizers--implications for radiation therapy. N. Engl. J. Med..

[48] Xing X., Cheng S.P., Huang J.B. (2024). Predicting angiogenesis in adrenal pheochromocytoma: The role of modified parameters from contrast-enhanced CT. Discov. Oncol..

[49] Castellano A., Bailo M., Cicone F., Carideo L., Quartuccio N., Mortini P., Falini A., Cascini G.L., Minniti G. (2021). Advanced Imaging Techniques for Radiotherapy Planning of Gliomas. Cancers.

[50] Corrias G., Lai E., Ziranu P., Mariani S., Donisi C., Liscia N., Saba G., Pretta A., Persano M., Fanni D. (2024). Prediction of Response to Anti-Angiogenic Treatment for Advanced Colorectal Cancer Patients: From Biological Factors to Functional Imaging. Cancers.

[51] Ciepla J., Smolarczyk R. (2024). Tumor hypoxia unveiled: Insights into microenvironment, detection tools and emerging therapies. Clin. Exp. Med..

[52] Bouleftour W., Rowinski E., Louati S., Sotton S., Wozny A.S., Moreno-Acosta P., Mery B., Rodriguez-Lafrasse C., Magne N. (2021). A Review of the Role of Hypoxia in Radioresistance in Cancer Therapy. Med. Sci. Monit..

[53] Wang H., Jiang H., Van De Gucht M., De Ridder M. (2019). Hypoxic Radioresistance: Can ROS Be the Key to Overcome It?. Cancers.

[54] Sorensen B.S., Horsman M.R. (2020). Tumor Hypoxia: Impact on Radiation Therapy and Molecular Pathways. Front. Oncol..

[55] Hanley R., Pagliari F., Garcia-Calderon D., Fernandes Guerreiro J., Genard G., Jansen J., Nistico C., Marafioti M.G., Tirinato L., Seco J. (2023). Radio-resistance of hypoxic tumors: Exploring the effects of oxygen and X-ray radiation on non-small lung cancer cell lines. Radiat. Oncol..

[56] Tian Y., Lei Y., Wang Y., Lai J., Wang J., Xia F. (2023). Mechanism of multidrug resistance to chemotherapy mediated by P-glycoprotein (Review). Int. J. Oncol..

[57] Bui B.P., Nguyen P.L., Lee K., Cho J. (2022). Hypoxia-Inducible Factor-1: A Novel Therapeutic Target for the Management of Cancer, Drug Resistance, and Cancer-Related Pain. Cancers.

[58] Xia Y., Jiang L., Zhong T. (2018). The role of HIF-1alpha in chemo-/radioresistant tumors. Onco Targets Ther..

[59] Scanlon S.E., Glazer P.M. (2015). Multifaceted control of DNA repair pathways by the hypoxic tumor microenvironment. DNA Repair.

[60] Bozward A.G., Warricker F., Oo Y.H., Khakoo S.I. (2021). Natural Killer Cells and Regulatory T Cells Cross Talk in Hepatocellular Carcinoma: Exploring Therapeutic Options for the Next Decade. Front. Immunol..

[61] Wang B., Zhao Q., Zhang Y., Liu Z., Zheng Z., Liu S., Meng L., Xin Y., Jiang X. (2021). Targeting hypoxia in the tumor microenvironment: A potential strategy to improve cancer immunotherapy. J. Exp. Clin. Cancer Res..

[62] Yang Y., Li C., Liu T., Dai X., Bazhin A.V. (2020). Myeloid-Derived Suppressor Cells in Tumors: From Mechanisms to Antigen Specificity and Microenvironmental Regulation. Front. Immunol..

[63] Sethumadhavan S., Silva M., Philbrook P., Nguyen T., Hatfield S.M., Ohta A., Sitkovsky M.V. (2017). Hypoxia and hypoxia-inducible factor (HIF) downregulate antigen-presenting MHC class I molecules limiting tumor cell recognition by T cells. PLoS ONE.

[64] Zhang W., Wang M., Ji C., Liu X., Gu B., Dong T. (2024). Macrophage polarization in the tumor microenvironment: Emerging roles and therapeutic potentials. Biomed. Pharmacother..

[65] Gillies R.J., Kinahan P.E., Hricak H. (2016). Radiomics: Images Are More than Pictures, They Are Data. Radiology.

[66] Beig N., Patel J., Prasanna P., Hill V., Gupta A., Correa R., Bera K., Singh S., Partovi S., Varadan V. (2018). Radiogenomic analysis of hypoxia pathway is predictive of overall survival in Glioblastoma. Sci. Rep..

[67] Shao Y., Cen H.S., Dhananjay A., Pawan S.J., Lei X., Gill I.S., D’Souza A., Duddalwar V.A. (2025). Radiogenomic correlation of hypoxia-related biomarkers in clear cell renal cell carcinoma. J. Cancer Res. Clin. Oncol..

[68] Muller J., Leger S., Zwanenburg A., Suckert T., Luhr A., Beyreuther E., von Neubeck C., Krause M., Lock S., Dietrich A. (2022). Radiomics-based tumor phenotype determination based on medical imaging and tumor microenvironment in a preclinical setting. Radiother. Oncol..

[69] Zheng D., Grandgenett P.M., Zhang Q., Baine M., Shi Y., Du Q., Liang X., Wong J., Iqbal S., Preuss K. (2024). radioGWAS links radiome to genome to discover driver genes with somatic mutations for heterogeneous tumor image phenotype in pancreatic cancer. Sci. Rep..

[70] El-Baz A., Beache G.M., Gimel’farb G., Suzuki K., Okada K., Elnakib A., Soliman A., Abdollahi B. (2013). Computer-aided diagnosis systems for lung cancer: Challenges and methodologies. Int. J. Biomed. Imaging.

[71] Brooks F.J., Grigsby P.W. (2014). The effect of small tumor volumes on studies of intratumoral heterogeneity of tracer uptake. J. Nucl. Med..

[72] Hong S., Hong S., Oh E., Lee W.J., Jeong W.K., Kim K. (2024). Development of a flexible feature selection framework in radiomics-based prediction modeling: Assessment with four real-world datasets. Sci. Rep..

[73] Majumder S., Katz S., Kontos D., Roshkovan L. (2024). State of the art: Radiomics and radiomics-related artificial intelligence on the road to clinical translation. BJR Open.

[74] Clark K., Vendt B., Smith K., Freymann J., Kirby J., Koppel P., Moore S., Phillips S., Maffitt D., Pringle M. (2013). The Cancer Imaging Archive (TCIA): Maintaining and operating a public information repository. J. Digit. Imaging.

[75] Aerts H.J., Velazquez E.R., Leijenaar R.T., Parmar C., Grossmann P., Carvalho S., Bussink J., Monshouwer R., Haibe-Kains B., Rietveld D. (2014). Decoding tumour phenotype by noninvasive imaging using a quantitative radiomics approach. Nat. Commun..

[76] Chau M., Vu H., Debnath T., Rahman M.G. (2025). A scoping review of automatic and semi-automatic MRI segmentation in human brain imaging. Radiography.

[77] Fedorov A., Beichel R., Kalpathy-Cramer J., Finet J., Fillion-Robin J.C., Pujol S., Bauer C., Jennings D., Fennessy F., Sonka M. (2012). 3D Slicer as an image computing platform for the Quantitative Imaging Network. Magn. Reson. Imaging.

[78] Yushkevich P.A., Yang G., Gerig G. ITK-SNAP: An interactive tool for semi-automatic segmentation of multi-modality biomedical images. Proceedings of the 2016 38th Annual International Conference of the IEEE Engineering in Medicine and Biology Society (EMBC).

[79] Ronneberger O., Fischer P., Brox T. U-Net: Convolutional Networks for Biomedical Image Segmentation. Proceedings of the Medical Image Computing and Computer-Assisted Intervention—MICCAI 2015.

[80] van Griethuysen J.J.M., Fedorov A., Parmar C., Hosny A., Aucoin N., Narayan V., Beets-Tan R.G.H., Fillion-Robin J.C., Pieper S., Aerts H. (2017). Computational Radiomics System to Decode the Radiographic Phenotype. Cancer Res..

[81] Shi Z., Traverso A., van Soest J., Dekker A., Wee L. (2019). Technical Note: Ontology-guided radiomics analysis workflow (O-RAW). Med. Phys..

[82] Bakas S., Akbari H., Sotiras A., Bilello M., Rozycki M., Kirby J.S., Freymann J.B., Farahani K., Davatzikos C. (2017). Advancing The Cancer Genome Atlas glioma MRI collections with expert segmentation labels and radiomic features. Sci. Data.

[83] Marsilla J., Weiss J., Ye X.Y., Welch M., Milosevic M., Lyng H., Hompland T., Bruheim K., Tadic T., Haibe-Kains B. (2024). A T2-weighted MRI-based radiomic signature for disease-free survival in locally advanced cervical cancer following chemoradiation: An international, multicentre study. Radiother. Oncol..

[84] El-Manzalawy Y., Hsieh T.Y., Shivakumar M., Kim D., Honavar V. (2018). Min-redundancy and max-relevance multi-view feature selection for predicting ovarian cancer survival using multi-omics data. BMC Med. Genomics.

[85] Zhong X., Li L., Jiang H., Yin J., Lu B., Han W., Li J., Zhang J. (2020). Cervical spine osteoradionecrosis or bone metastasis after radiotherapy for nasopharyngeal carcinoma? The MRI-based radiomics for characterization. BMC Med. Imaging.

[86] Yuan Y., Tan L., Wang L., Zou D., Liu J., Lu X., Fu D., Wang G., Wang L., Wang Z. (2022). The Expression Pattern of Hypoxia-Related Genes Predicts the Prognosis and Mediates Drug Resistance in Colorectal Cancer. Front. Cell Dev. Biol..

[87] Zhou C., Jia H., Jiang N., Zhao J., Nan X. (2024). Establishment of Chemotherapy Prediction Model Based on Hypoxia-Related Genes for Oral Cancer. J. Cancer.

[88] Wang Q., Chang Z., Liu X., Wang Y., Feng C., Ping Y., Feng X. (2024). Predictive Value of Machine Learning for Platinum Chemotherapy Responses in Ovarian Cancer: Systematic Review and Meta-Analysis. J. Med. Internet Res..

[89] Guan X., Du Y., Ma R., Teng N., Ou S., Zhao H., Li X. (2023). Construction of the XGBoost model for early lung cancer prediction based on metabolic indices. BMC Med. Inform. Decis. Mak..

[90] Huang Y., Fan J., Li Y., Fu S., Chen Y., Wu J. (2021). Imaging of Tumor Hypoxia with Radionuclide-Labeled Tracers for PET. Front. Oncol..

[91] Tanaka S., Kadoya N., Sugai Y., Umeda M., Ishizawa M., Katsuta Y., Ito K., Takeda K., Jingu K. (2022). A deep learning-based radiomics approach to predict head and neck tumor regression for adaptive radiotherapy. Sci. Rep..

[92] Manescu P., Geradts J., Fernández-Reyes D. (2023). Deep learning-based detection of morphological features associated with hypoxia in H&E breast cancer whole slide images. arXiv.

[93] Pigat L., Geisler B.P., Sheikhalishahi S., Sander J., Kaspar M., Schmutz M., Rohr S.O., Wild C.M., Goss S., Zaghdoudi S. (2024). Predicting Hypoxia Using Machine Learning: Systematic Review. JMIR Med. Inform..

[94] de Vries B.M., Zwezerijnen G.J.C., Burchell G.L., van Velden F.H.P., Menke-van der Houven van Oordt C.W., Boellaard R. (2023). Explainable artificial intelligence (XAI) in radiology and nuclear medicine: A literature review. Front. Med..

[95] Nielsen T., Wittenborn T., Horsman M.R. (2012). Dynamic Contrast-Enhanced Magnetic Resonance Imaging (DCE-MRI) in Preclinical Studies of Antivascular Treatments. Pharmaceutics.

[96] Prasad P.V., Li L.P., Hack B., Leloudas N., Sprague S.M. (2023). Quantitative Blood Oxygenation Level Dependent Magnetic Resonance Imaging for Estimating Intra-renal Oxygen Availability Demonstrates Kidneys Are Hypoxemic in Human CKD. Kidney Int. Rep..

[97] O’Connor J.P.B., Robinson S.P., Waterton J.C. (2019). Imaging tumour hypoxia with oxygen-enhanced MRI and BOLD MRI. Br. J. Radiol..

[98] Bodalal Z., Bogveradze N., Ter Beek L.C., van den Berg J.G., Sanders J., Hofland I., Trebeschi S., Groot Lipman K.B.W., Storck K., Hong E.K. (2023). Radiomic signatures from T2W and DWI MRI are predictive of tumour hypoxia in colorectal liver metastases. Insights Imaging.

[99] Sersa I., Bajd F., Savarin M., Jesenko T., Cemazar M., Sersa G. (2018). Multiparametric High-Resolution MRI as a Tool for Mapping of Hypoxic Level in Tumors. Technol. Cancer Res. Treat..

[100] Bekaert L., Valable S., Lechapt-Zalcman E., Ponte K., Collet S., Constans J.M., Levallet G., Bordji K., Petit E., Branger P. (2017). [18F]-FMISO PET study of hypoxia in gliomas before surgery: Correlation with molecular markers of hypoxia and angiogenesis. Eur. J. Nucl. Med. Mol. Imaging.

[101] Thorwarth D., Welz S., Monnich D., Pfannenberg C., Nikolaou K., Reimold M., La Fougere C., Reischl G., Mauz P.S., Paulsen F. (2019). Prospective Evaluation of a Tumor Control Probability Model Based on Dynamic (18)F-FMISO PET for Head and Neck Cancer Radiotherapy. J. Nucl. Med..

[102] Bourigault P., Skwarski M., Macpherson R.E., Higgins G.S., McGowan D.R. (2022). Timing of hypoxia PET/CT imaging after 18F-fluoromisonidazole injection in non-small cell lung cancer patients. Sci. Rep..

[103] Perez R.C., Kim D., Maxwell A.W.P., Camacho J.C. (2023). Functional Imaging of Hypoxia: PET and MRI. Cancers.

[104] Qi Q., Yeung T.P., Lee T.Y., Bauman G., Crukley C., Morrison L., Hoffman L., Yartsev S. (2016). Evaluation of CT Perfusion Biomarkers of Tumor Hypoxia. PLoS ONE.

[105] Feng S., Xia T., Ge Y., Zhang K., Ji X., Luo S., Shen Y. (2022). Computed Tomography Imaging-Based Radiogenomics Analysis Reveals Hypoxia Patterns and Immunological Characteristics in Ovarian Cancer. Front. Immunol..

[106] Wang P., Luo Z., Luo C., Wang T. (2024). Application of a Comprehensive Model Based on CT Radiomics and Clinical Features for Postoperative Recurrence Risk Prediction in Non-small Cell Lung Cancer. Acad. Radiol..

[107] Tran A., Koh T.S., Prawira A., Ho R.Z.W., Le T.B.U., Vu T.C., Hartano S., Teo X.Q., Chen W.C., Lee P. (2021). Dynamic Contrast-Enhanced Magnetic Resonance Imaging as Imaging Biomarker for Vascular Normalization Effect of Infigratinib in High-FGFR-Expressing Hepatocellular Carcinoma Xenografts. Mol. Imaging Biol..

[108] Garcia-Figueiras R., Baleato-Gonzalez S., Luna A., Padhani A.R., Vilanova J.C., Carballo-Castro A.M., Oleaga-Zufiria L., Vallejo-Casas J.A., Marhuenda A., Gomez-Caamano A. (2024). How Imaging Advances Are Defining the Future of Precision Radiation Therapy. Radiographics.

[109] Lim W.H., Park J.S., Park J., Choi S.H. (2021). Assessing the reproducibility of high temporal and spatial resolution dynamic contrast-enhanced magnetic resonance imaging in patients with gliomas. Sci. Rep..

[110] Hernandez-Agudo E., Mondejar T., Soto-Montenegro M.L., Megias D., Mouron S., Sanchez J., Hidalgo M., Lopez-Casas P.P., Mulero F., Desco M. (2016). Monitoring vascular normalization induced by antiangiogenic treatment with (18)F-fluoromisonidazole-PET. Mol. Oncol..

[111] Mirus M., Tokalov S.V., Abramyuk A., Heinold J., Prochnow V., Zophel K., Kotzerke J., Abolmaali N. (2019). Noninvasive assessment and quantification of tumor vascularization using [18F]FDG-PET/CT and CE-CT in a tumor model with modifiable angiogenesis-an animal experimental prospective cohort study. EJNMMI Res..

[112] Moses W.W. (2011). Fundamental Limits of Spatial Resolution in PET. Nucl. Instrum. Methods Phys. Res. A.

[113] Garcia-Figueiras R., Goh V.J., Padhani A.R., Baleato-Gonzalez S., Garrido M., Leon L., Gomez-Caamano A. (2013). CT perfusion in oncologic imaging: A useful tool?. AJR Am. J. Roentgenol..

[114] Jain R., Ellika S.K., Scarpace L., Schultz L.R., Rock J.P., Gutierrez J., Patel S.C., Ewing J., Mikkelsen T. (2008). Quantitative estimation of permeability surface-area product in astroglial brain tumors using perfusion CT and correlation with histopathologic grade. AJNR Am. J. Neuroradiol..

[115] Tachiiri T., Nishiofuku H., Maeda S., Sato T., Toyoda S., Matsumoto T., Chanoki Y., Minamiguchi K., Taiji R., Kunichika H. (2023). Vascular Normalization Caused by Short-Term Lenvatinib Could Enhance Transarterial Chemoembolization in Hepatocellular Carcinoma. Curr. Oncol..

[116] Crispin-Ortuzar M., Apte A., Grkovski M., Oh J.H., Lee N.Y., Schoder H., Humm J.L., Deasy J.O. (2018). Predicting hypoxia status using a combination of contrast-enhanced computed tomography and [(18)F]-Fluorodeoxyglucose positron emission tomography radiomics features. Radiother. Oncol..

[117] Tunali I., Tan Y., Gray J.E., Katsoulakis E., Eschrich S.A., Saller J., Aerts H., Boyle T., Qi J., Guvenis A. (2021). Hypoxia-Related Radiomics and Immunotherapy Response: A Multicohort Study of Non-Small Cell Lung Cancer. JNCI Cancer Spectr..

[118] Carles M., Fechter T., Grosu A.L., Sorensen A., Thomann B., Stoian R.G., Wiedenmann N., Ruhle A., Zamboglou C., Ruf J. (2021). (18)F-FMISO-PET Hypoxia Monitoring for Head-and-Neck Cancer Patients: Radiomics Analyses Predict the Outcome of Chemo-Radiotherapy. Cancers.

[119] Muzi M., Wolsztynski E., Fink J.R., O’Sullivan J.N., O’Sullivan F., Krohn K.A., Mankoff D.A. (2020). Assessment of the Prognostic Value of Radiomic Features in (18)F-FMISO PET Imaging of Hypoxia in Postsurgery Brain Cancer Patients: Secondary Analysis of Imaging Data from a Single-Center Study and the Multicenter ACRIN 6684 Trial. Tomography.

[120] Ogbonnaya C.N., Alsaedi B.S.O., Alhussaini A.J., Hislop R., Pratt N., Nabi G. (2023). Radiogenomics Reveals Correlation between Quantitative Texture Radiomic Features of Biparametric MRI and Hypoxia-Related Gene Expression in Men with Localised Prostate Cancer. J. Clin. Med..

[121] Shui L., Ren H., Yang X., Li J., Chen Z., Yi C., Zhu H., Shui P. (2020). The Era of Radiogenomics in Precision Medicine: An Emerging Approach to Support Diagnosis, Treatment Decisions, and Prognostication in Oncology. Front. Oncol..

[122] Singh G., Manjila S., Sakla N., True A., Wardeh A.H., Beig N., Vaysberg A., Matthews J., Prasanna P., Spektor V. (2021). Radiomics and radiogenomics in gliomas: A contemporary update. Br. J. Cancer.

[123] Huang W., Jiang Y., Xiong W., Sun Z., Chen C., Yuan Q., Zhou K., Han Z., Feng H., Chen H. (2022). Noninvasive imaging of the tumor immune microenvironment correlates with response to immunotherapy in gastric cancer. Nat. Commun..

[124] Cho H., Ackerstaff E., Carlin S., Lupu M.E., Wang Y., Rizwan A., O’Donoghue J., Ling C.C., Humm J.L., Zanzonico P.B. (2009). Noninvasive multimodality imaging of the tumor microenvironment: Registered dynamic magnetic resonance imaging and positron emission tomography studies of a preclinical tumor model of tumor hypoxia. Neoplasia.

[125] Gertsenshteyn I., Epel B., Giurcanu M., Barth E., Lukens J., Hall K., Martinez J.F., Grana M., Maggio M., Miller R.C. (2023). Absolute oxygen-guided radiation therapy improves tumor control in three preclinical tumor models. Front. Med..

[126] Meissner A.K., Gutsche R., Galldiks N., Kocher M., Junger S.T., Eich M.L., Nogova L., Araceli T., Schmidt N.O., Ruge M.I. (2023). Radiomics for the non-invasive prediction of PD-L1 expression in patients with brain metastases secondary to non-small cell lung cancer. J. Neurooncol..

[127] Li Y., Zhao L., Li X.F. (2021). Targeting Hypoxia: Hypoxia-Activated Prodrugs in Cancer Therapy. Front. Oncol..

[128] McDonald P.C., Winum J.Y., Supuran C.T., Dedhar S. (2012). Recent developments in targeting carbonic anhydrase IX for cancer therapeutics. Oncotarget.

[129] Sanduleanu S., Jochems A., Upadhaya T., Even A.J.G., Leijenaar R.T.H., Dankers F., Klaassen R., Woodruff H.C., Hatt M., Kaanders H. (2020). Non-invasive imaging prediction of tumor hypoxia: A novel developed and externally validated CT and FDG-PET-based radiomic signatures. Radiother. Oncol..

[130] Brenner A.J., Floyd J., Fichtel L., Michalek J., Kanakia K.P., Huang S., Reardon D., Wen P.Y., Lee E.Q. (2021). Phase 2 trial of hypoxia activated evofosfamide (TH302) for treatment of recurrent bevacizumab-refractory glioblastoma. Sci. Rep..

[131] Welz S., Paulsen F., Pfannenberg C., Reimold M., Reischl G., Nikolaou K., La Fougere C., Alber M., Belka C., Zips D. (2022). Dose escalation to hypoxic subvolumes in head and neck cancer: A randomized phase II study using dynamic [(18)F]FMISO PET/CT. Radiother. Oncol..

[132] Zheng J., Xu S., Wang G., Shi Y. (2024). Applications of CT-based radiomics for the prediction of immune checkpoint markers and immunotherapeutic outcomes in non-small cell lung cancer. Front. Immunol..

[133] Ma L., Guo H., Zhao Y., Liu Z., Wang C., Bu J., Sun T., Wei J. (2024). Liquid biopsy in cancer current: Status, challenges and future prospects. Signal Transduct. Target. Ther..

[134] Stejskal P., Goodarzi H., Srovnal J., Hajduch M., van‘t Veer L.J., Magbanua M.J.M. (2023). Circulating tumor nucleic acids: Biology, release mechanisms, and clinical relevance. Mol. Cancer.

[135] Zhou L., Mao C., Fu T., Ding X., Bertolaccini L., Liu A., Zhang J., Li S. (2024). Development of an AI model for predicting hypoxia status and prognosis in non-small cell lung cancer using multi-modal data. Transl. Lung Cancer Res..

[136] Captier N., Lerousseau M., Orlhac F., Hovhannisyan-Baghdasarian N., Luporsi M., Woff E., Lagha S., Salamoun Feghali P., Lonjou C., Beaulaton C. (2025). Integration of clinical, pathological, radiological, and transcriptomic data improves prediction for first-line immunotherapy outcome in metastatic non-small cell lung cancer. Nat. Commun..

[137] Limkin E.J., Sun R., Dercle L., Zacharaki E.I., Robert C., Reuze S., Schernberg A., Paragios N., Deutsch E., Ferte C. (2017). Promises and challenges for the implementation of computational medical imaging (radiomics) in oncology. Ann. Oncol..

